# Clinical study of right ventricular longitudinal strain for assessing right ventricular dysfunction and hemodynamics in pulmonary hypertension

**DOI:** 10.1097/MD.0000000000005668

**Published:** 2016-12-16

**Authors:** Yidan Li, Yidan Wang, Xiaoguang Ye, Lingyun Kong, Weiwei Zhu, Xiuzhang Lu

**Affiliations:** Department of Echocardiography, Heart Center, Beijing Chao Yang Hospital, Capital Medical University, Beijing, China.

**Keywords:** hemodynamics, pulmonary hypertension, right, speckle tracking imaging, ventricular function

## Abstract

This study aimed to appraise the application of right ventricular longitudinal strain for assessing right ventricular dysfunction and severe hemodynamic changes in pulmonary hypertension. The study included 53 patients clinically diagnosed with PH. Tissue Doppler–derived tricuspid lateral annular systolic velocity (s’), early diastolic peak velocity (e’), late diastolic peak velocity (a’), tricuspid annular plane systolic excursion (TAPSE), RV index of myocardial performance (RIMP), and right ventricular fractional area change (FAC) were determined. The STI parameter was RV free wall longitudinal peak systolic strain (RV LPSS). The patients were assigned into two groups based on a RV LPSS value of − 19%.

RV LPSS, s’, TAPSE, RIMP, FAC, a’ and e’/a’ showed significant differences. PH patients with an RV LPSS≥ −19% exhibited a lower RV function (*P* < 0.05). RV LPSS was negatively correlated with TAPSE (*r* = −0.326, *P* < 0.05) and FAC (*r* = −0.495, *P* < 0.001) and positively correlated with RIMP (*r* = 0.508, *P* < 0.001). The optimal cut-off value of RV LPSS to reveal an mPAP ≥ 45 mmHg defined based on the receiver operating characteristic curve analysis was − 19.26% with a sensitivity of 83.9% and a specificity of 73.4%.

Distinguishing the degree of RV dysfunction by 2D-STI may help physicians to determine the state of cardiac function and degree of PH in patients and offer a basis for subsequent clinical diagnosis and therapy. Our study demonstrates the superiority of RV LPSS for uncovering severe PH over the traditional echocardiographic parameters.

## 1 Introduction

Right ventricular (RV) function is a valuable predictor of prognosis for patients with pulmonary hypertension (PH), because it is well correlated to clinical consequences, severity of illness, and the quality of patient's daily life. Although pulmonary load is a significant determinant of RV systolic function, there remains great variability in RV adaptation to PH. Therefore, correct assessment of RV function is relevant in the clinical managing of these patients. PH is a pathophysiological disorder that is present in multiple clinical manifestations and complicates a variety of cardiovascular and respiratory diseases.^[1]^ PH is defined as an increase in the mean pulmonary arterial pressure (mPAP) ≥25 mm Hg at rest as determined by right heart catheterization (RHC), which is required to confirm the diagnosis of PH, to evaluate the severity of hemodynamic impairment, and to undertake vasoreactivity testing of the pulmonary circulation in selected patients. RHC showed a low morbidity (1.1%) and mortality (0.055%) when conducted at professional centers.^[2]^ Transthoracic echocardiography is used to image the pulmonary artery pressure in the heart and to estimate the PAP from the impact of continuous wave Doppler measurements. With regard to PH, echocardiography is not sufficient for determining the appropriate treatment decision, and cardiac catheterization is required.^[1]^ However, RHC is invasive and impractical for serial assessment. Therefore, a noninvasive method to detect pulmonary hemodynamics is needed.

Two-dimensional (2D) echocardiography is the widely utilized means for appraisal of the RV function in clinical practice. Several studies showed the application of a number of echocardiographic parameters in the evaluation of RV function, including tricuspid annular plane systolic excursion (TAPSE), the tricuspid annular plane systolic velocity (s′),^[3]^ RV fractional area change (RVFAC), and RV myocardial performance index (RIMP).^[4,5]^ However, in those studies, the free lateral wall of the RV was appraised with M-Mode and tissue Doppler imaging only in 1 plane and was employed as a surrogate for RV function. Hence, the present parameters that are measured by echocardiographic techniques only under a simple algorithm are not suitable for calculating RV function.^[6]^

Myocardial deformation evaluated by strain is more valuable than wall motion analysis (velocity and displacement) in detecting regional myocardial abnormalities. Recently, several studies demonstrated that two-dimensional speckle tracking strain imaging (2D-STI) is a functional modality in the appraisal of RV function.^[7,8]^ Longitudinal strain (LS) is independent of global cardiac movement, thus allowing a quantitative analysis of regional myocardial deformation in different RV segments.^[9]^ A previous study demonstrated that RV longitudinal strain as assessed with speckle tracking strain imaging was severely impaired in patients with PH, and that it was inversely correlated with systolic pulmonary arterial pressure (SPAP) and RV dimensions.^[8]^

In this study, we measured the RV myocardial longitudinal strain (RV LPSS) of PH patients using 2D-STI and analyzed the correlation between RV LPSS and the change in RV function. We also detected severe PH (mPAP ≥45 mm Hg) by RV LPSS and investigated the ability of RV LPSS to predict severe PH in patients using 2D-STI.

## 2 Patients and methods

### 2.1 Study patients

A total of 76 consecutive patients with PH who visited our Hospital from August 2013 to December 2014 were enrolled in our study. Diagnosis of PH was defined as a mean PAP ≥25 mm Hg assessed with RHC, or SPAP ≥36 mm Hg estimated with echocardiography.^[10]^ The exclusion criteria included: RHC was not performed; poor echocardiography image quality; coronary artery disease, moderate to severe aortic and/or mitral valval heart disease, left-sided heart failure, and atrial fibrillation/flutter. All patients were extensively screened to confirm the diagnosis of PH. Invasive measurement of pulmonary pressures was performed with right heart catheterization when SPAP was 36 mm Hg. After the application of the exclusion criteria, 53 cases with PH were included in our study. The study cohort included 25 patients with chronic thromboembolic PH, 18 with idiopathic PH, 6 with PH associated with connective tissue disease, 2 with PH associated with congenital heart disease, and 2 with heritable PH. This study was in compliance with the guidelines of the Declaration of Helsinki. The Ethics Committee of our Hospital approved this study. All participants provided informed consent for participation in this study.

### 2.2 Echocardiography

All echocardiography was performed with an Artida ultrasound system (Toshiba Medical Systems, Tochigi, Japan). Images were obtained with the patient in the left lateral decubitus position. M-mode was employed to assess the TAPSE. The M-mode cursor was positioned through the lateral side of the tricuspid annulus in such a way that the position of annulus changed along with the cursor. The systolic displacement of annular was assessed from end-diastole to end-systole. The tricuspid annular systolic velocity (s′) was measured in the apical 4-chamber view though tissue Doppler imaging. The isovolumic acceleration of the RV was computed as the peak isovolumic myocardial velocity divided by the time to peak velocity, as assessed by tissue Doppler imaging at the lateral tricuspid annulus. The RVFAC was computed as reported (ref): (RV diastolic area–RV systolic area)/RV diastolic area × 100%. The RV diastolic and systolic areas were acquired from the apical 4-chamber view. RVFAC = the right ventricular index of myocardial performance (RIMP) was the isovolumic time divided by the ejecting time, which was evaluated in the same pulsed. The isovolumic time was computed by subtracting the ejecting time from the tricuspid closure time. The ratio of the RV transverse diameter to the left ventricular transverse diameter was calculated at the basal segment at the end of diastole in the apical 4-chamber view.

### 2.3 Two-dimensional speckle tracking analysis

**Figure 1:**
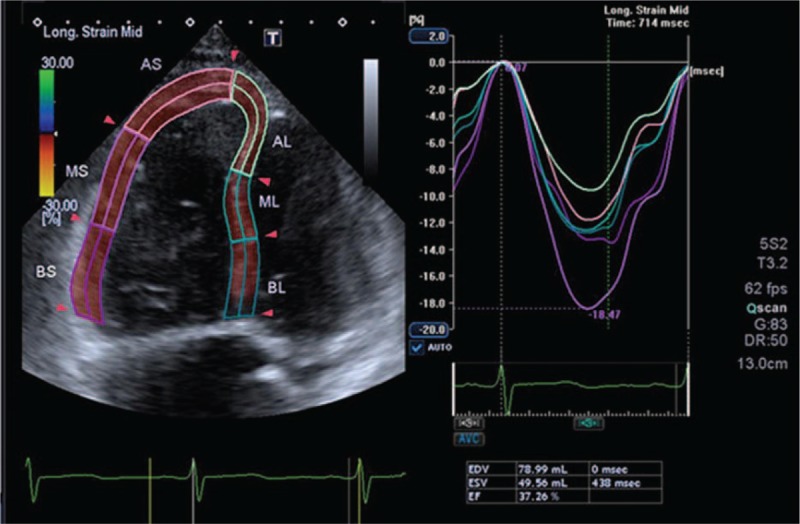
Measurement of RV LPSS by using 2D-STI. Right ventricular free-wall longitudinal speckle tracking strain (RV-free) was obtained by averaging the basal, middle, and apical lateral peak systolic strains along the entire right ventricle using the RV-focused view. 2D-STI = two-dimensional speckle tracking strain imaging, RV = right ventricular, RV LPSS = RV free wall longitudinal peak systolic strain.

Three cardiac cycles were used to continuously image the right ventricle as the center of the 4 chambers, with a frame rate >60 frames/s. With 2D-STI performed online with dedicated software, the longitudinal strain of RV free wall was evaluated from an RV-focused apical 4-chamber view. Generally, we traced a region of interest by point-and-click approach on the endocardium at end-diastole in RV from the RV-focused view. A second larger region of interest was further produced and manually fine-tuned near the epicardium. The region of interest was carefully adjusted using visual assessment to assure that every segment was tracked perfectly. The right ventricle was partitioned into 6 standard segments at 3 levels (i.e., the basal, middle, and apical levels), correspondingly generating 6 time-strain curves (Fig. 1). RV LPSS was evaluated in the basal, midventricular, and apical segments of the RV free wall and calculated as the average of the 3 segments. RV LPSS is determined as the percentage of myocardial shortening relative to the original length and is conventionally presented as a negative value. Therefore, the more negative the value of RV LPSS, the more preserved is the shortening.

### 2.4 Hemodynamic measurements

Hemodynamic measurements were obtained in all patients via right-heart cardiac catheterization. The measured indices were mPAP, pulmonary capillary wedge pressure (PCWP), and pulmonary vascular resistance (PVR). Cardiac output was determined by the Fick method. An investigator blinded to the echocardiographic measurements evaluated the pressure.

### 2.5 Statistical analyses

Continuous variables are presented as mean ± standard deviation. Normality was evaluated using the 1-sample Kolmogorov–Smirnov test. Linear regression analysis was used to evaluate the relationships between the 2 variables. The abilities of RV LPSS, FAC, RIMP, s′, and TAPSE to detect severe PH were appraised with receiver operating characteristic (ROC) curves to yield optimal values for an area under the curve (AUC). ROC curves were established to confirm the optimal cut-off value of RV LPSS that indicated elevated mPAP. The optimal cut-off value was determined as the point nearest to 1 in the top left corner. Reliability was assessed as the absolute difference in strain measurements between the 2 observers divided by the mean of both measurements and expressed as a percentage. Interobserver and intraobserver intraclass correlation coefficients (ICCs) for quantitative measurement of RV LPSS were calculated in a 2-way mixed model with 95% confidence intervals (CIs). The agreement between interobserver and intraobserver reliability analyses was tested by the Bland–Altman method. A *P* value less than 0.05 was considered statistically significant. All statistical analysis and graphic presentation were performed with the statistical software SPSS (version 17.0 for Windows; SPSS, Chicago, IL).

## 3 Results

### 3.1 Patients characteristics

A total of 53 consecutive patients with PH were registered in this study including 25 men and 28 women with a mean age of 53.5 ± 13.1 years and an average body mass index of 22.77 ± 5.37 kg/m^2^ (Table 1).

**Table 1:**
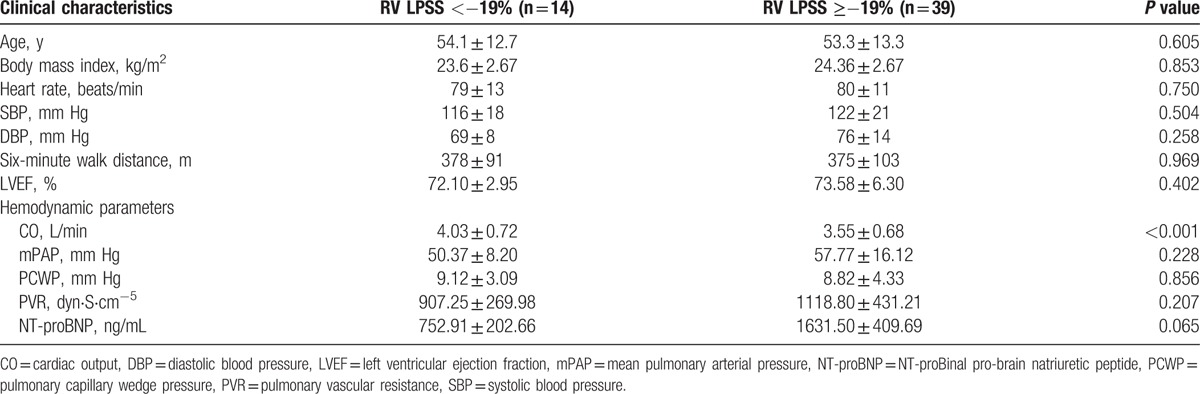
Comparison of clinical characteristics of participants in the 2 groups of this study.

On the basis of a prespecified cut-off value of RV LPSS, the patient population was categorized into 2 groups: Group I: patients with RV LPSS < −19% (more preserved RV myocardial shortening) (n = 14) and Group II: patients with RV LPSS ≥−19% (more impaired RV myocardial shortening) (n = 39).^[11,12]^These 2 groups were comparable with respect to age, body surface area, and so on (Table 1).

### 3.2 RV measurement

The comparison of echocardiography parameters for evaluating right ventricular between 2 groups contained 2 parts: RV structure and RV function. Both groups were comparable in terms of left ventricular transverse diameter (LVTD), inner diameter of main pulmonary artery (D_MPA_), inner diameter of left arterial branch (D_LPA_), and inner diameter of right pulmonary arterial branch (D_RPA_). Only right ventricular transverse diameter (RVTD) and RVTD/ LVTD showed significant differences between Group I and II (*P* *<*0.05). For right ventricular function, RV LPSS, TAPSE, s′, RIMP, FAC, a′, and e′/a′ had significantly statistic differences between Group I and II (*P* *<*0.05). Patients with RV LPSS ≥−19% exhibited lower RV function (Table 2).

**Table 2:**
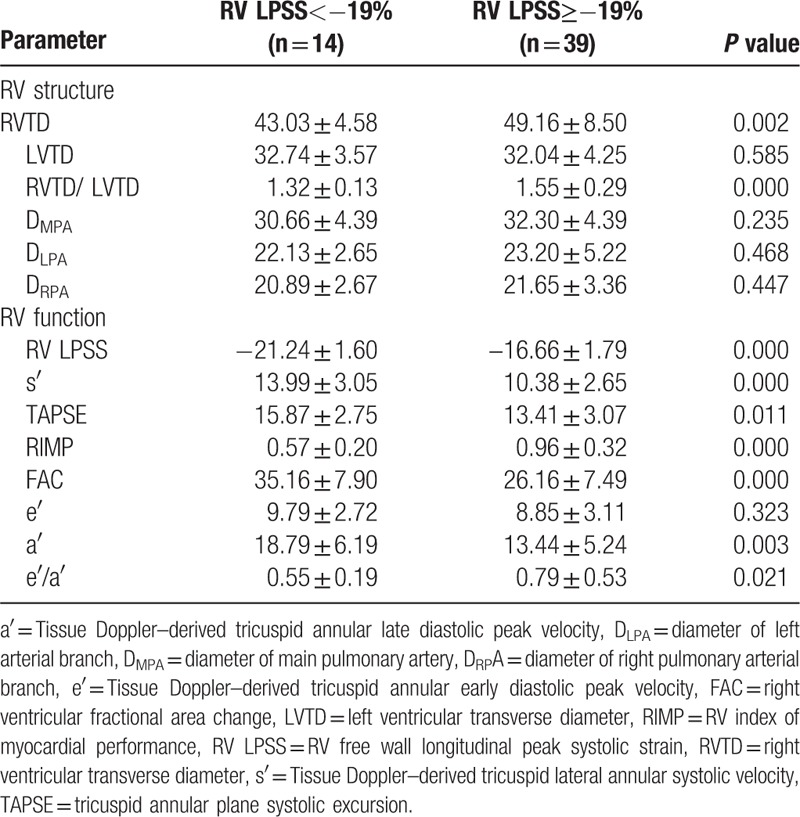
Echocardiographic parameters of participants in the group with RV LPSS < or ≥ −19%.

### 3.3 Correlation analysis of RV LPSS and RV function parameters

**Figure 2:**
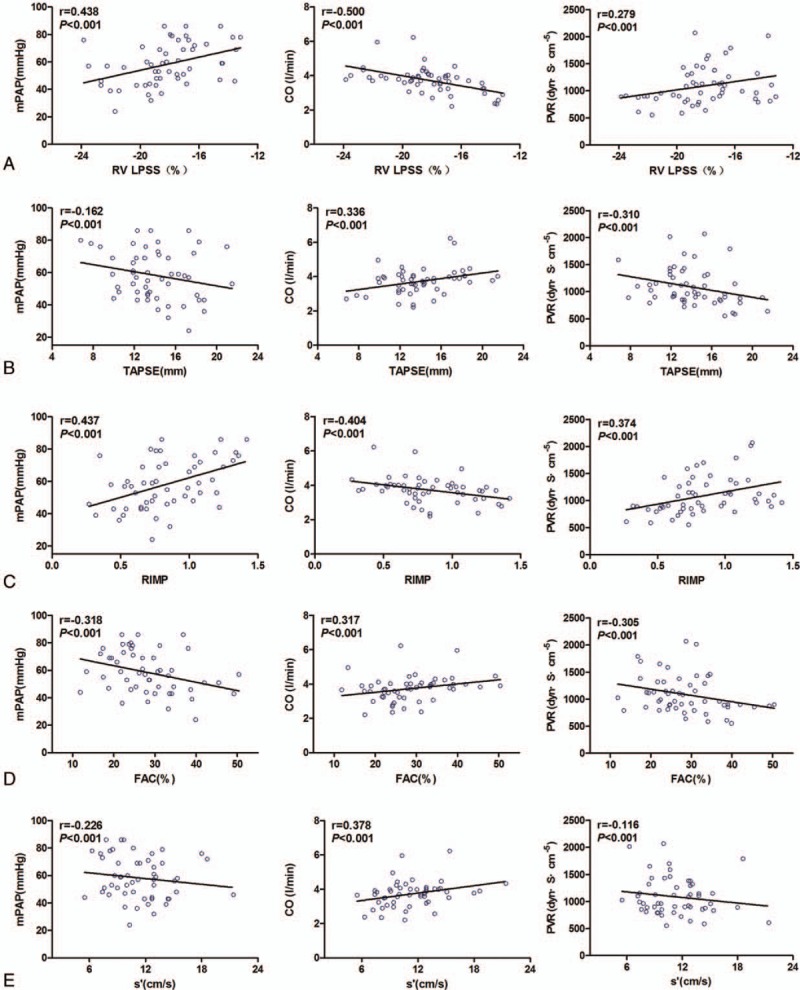
Correlations between the echo parameters and RHC parameters. A, Correlations between RV LPSS and mPAP, PVR, and CO. B, Correlations between TAPSE and mPAP, PVR, and CO. C, Correlations between RIMP and mPAP, PVR, and CO. D, Correlations between FAC and mPAP, PVR, and CO. E, Correlations between s′ and mPAP, PVR, and CO. CO = cardiac output, FAC = fractional area change, mPAP = mean pulmonary arterial pressure, PVR = pulmonary vascular resistance, RIMP = right ventricular index of myocardial performance, RHC = right heart catheterisation, RV LPSS = right ventricular free wall longitudinal peak systolic strain, s′ = tricuspid lateral annular systolic velocity, TAPSE = tricuspid annular plane systolic excursion.

**Figure 3:**
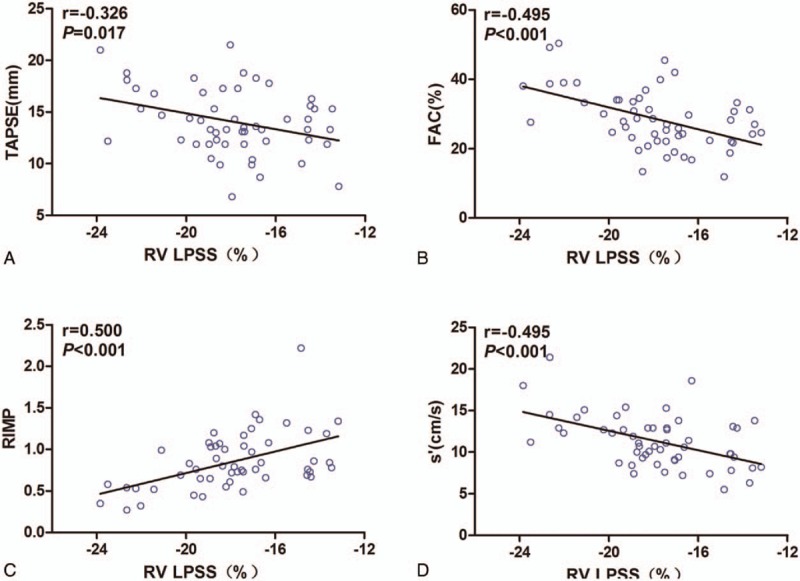
Correlation between RV LPSS and RV function parameters. A, Correlation between RV LPSS and TAPSE. B, Correlation between RV LPSS and FAC. C, Correlation between RV LPSS and RIMP. D, Correlation between RV LPSS and s′. FAC = fractional area change, RIMP = right ventricular index of myocardial performance, RV = right ventricular, RV LPSS = right ventricular free wall longitudinal peak systolic strain, s′ = tricuspid lateral annular systolic velocity, TAPSE = tricuspid annular plane systolic excursion.

**Figure 4:**
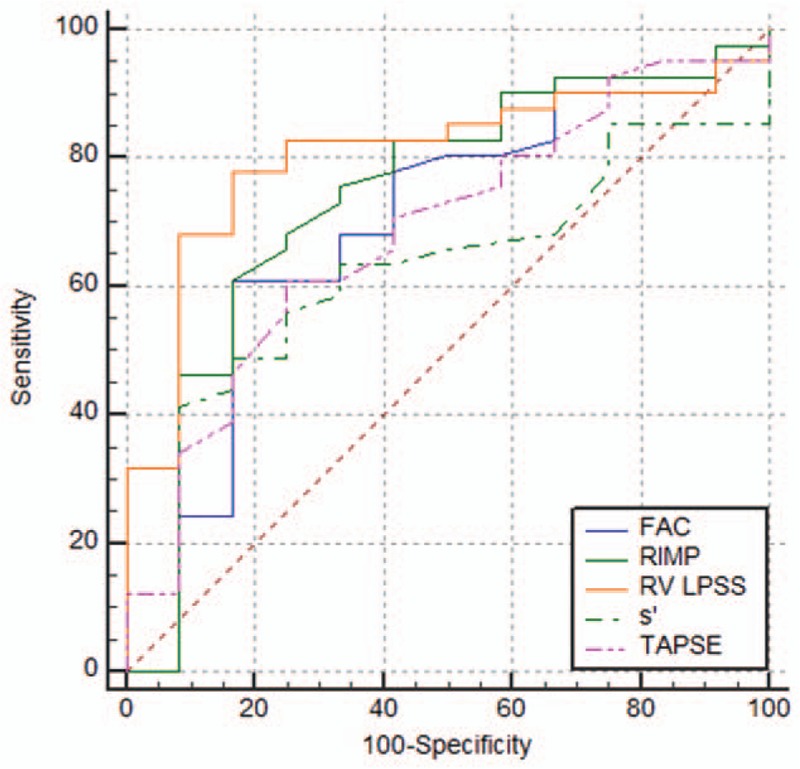
Echocardiographic parameters (FAC, RIMP, RV LPSS, s′, and TAPSE) were useful for diagnosing mPAP ≥45 mm Hg.

As shown in Figure 2A to E, correlations were observed between the echo parameters (RV LPSS, TAPSE, RIMP, FAC, and s′) and RHC parameters (mPAP, PVR, and CO). The RV LPSS significantly correlated with TAPSE (*r* = −0.326; *P* =0.017), FAC (*r* = −0.495, *P* <0.001)), RIMP (*r* = 0.5, *P* <0.001)), and s′ (*r* = −0.495, *P* <0.001) (Fig. 3). As shown in Table 3, ROC curves were used to obtain AUC values for the ability of RV LPSS, FAC, RIMP, s′, and TAPSE to detect severe PH (mPAP ≥45 mmHg). The most favorable cut-off value of RV LPSS for uncovering mPAP ≥45 mm Hg was −19.26% with a sensitivity of 83.9% and a specificity of 73.4% (Fig. 4).

**Table 3:**
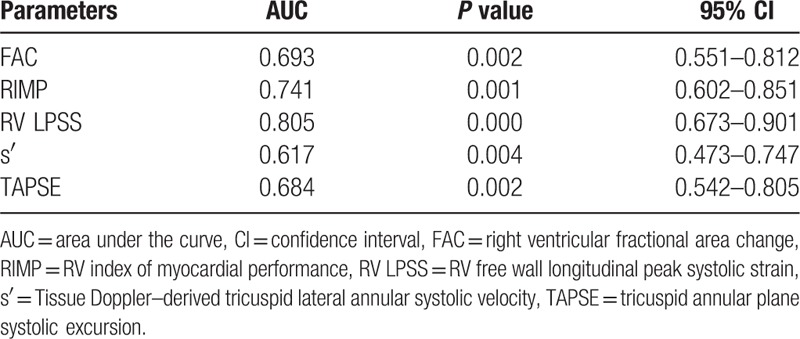
Area under the curve (AUC) for the ability of RV LPSS, FAC, RIMP, s′, and TAPSE to detect severe PH defined by a mPAP ≥45 mm Hg.

### 3.4 Reliability studies of RV strain

**Figure 5:**
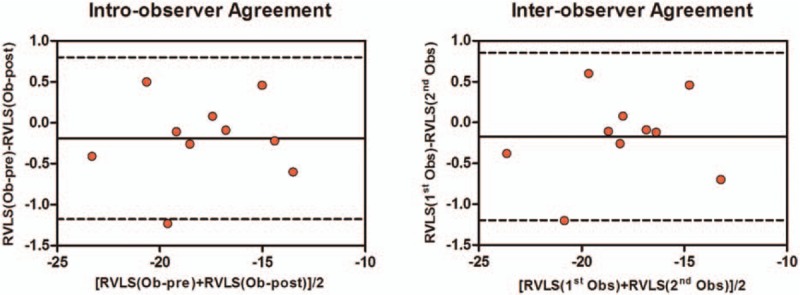
Reliability studies of RV strain. The inter- and intraobserver reliability analysis of RV LPSS was assessed in 10 subjects. RV = right ventricular, RV LPSS = right ventricular free wall longitudinal peak systolic strain.

The interobserver reliability for RV strain was assessed using measurements by different observers in a series of 10 patients and then measured by examiners blinded to the patient types. One of 2 observers was an expert in echocardiography. The intraobserver reliability of RV strain over time was investigated in a series of 10 patients (Table 4, Fig. 5).

**Table 4:**
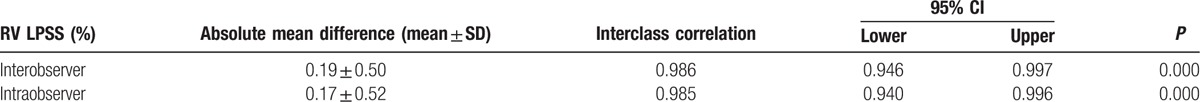
Inter- and intraobserver reliability of measurements (n = 10).

## 4 Discussion

PH is a catastrophic disease, in which both vasoconstriction and vascular remodeling occur and give rise to a gradual increase in pulmonary vascular resistance. Although the initial pathological changes in PH involve the pulmonary vasculature, the prognosis of PH patients was closely linked to RV function.^[13]^ Independent of the type of PH, the patients will progress to refractory right heart failure eventually. The function of right ventricle is a significant predictor of prognosis, because it is well correlated to severity of illness, clinical consequences, and the quality of patient's daily life. Generally speaking, severe PH was defined by a mPAP ≥35 mm Hg.^[14,15]^ However, an mPAP >45 mm Hg or even higher can lead to poor prognosis in patients with PH, and clinical combination therapy and/or surgical lung transplantation surgery is more likely to be used for the treatment.^[16–18]^

The impaired RV function contributes significantly to the morbidity and mortality of patients with heart and lung diseases. However, the best way to evaluate RV function has not been determined. One of the reasons is the excessive focus on the appraisal of left ventricular function. Another reason is the lack of ultrasound techniques for right heart imaging. It is therefore imperative to find a feasible way to evaluate right ventricular function, which may detect right heart failure in the subclinical stage before progression to right heart failure. Two-dimensional speckle tracking is a novel technology that may be used to assess the RV function. It provides angle-independent assessment of regional myocardial deformation and does not rely on geometrical assumptions.^[19]^ The right ventricular wall is thin, and the right ventricular myocardium is mainly composed of epicardia longitudinal myocardium and endocardial annular myocardium. Moreover, the longitudinal myocardium plays a main role in right ventricular systolic and diastolic processes. Therefore, studies of STI for evaluating right ventricular function have mainly focused on the longitudinal direction of myocardial motion.^[20]^

In the current study, no significant differences in age, tricuspid regurgitation area, SPAP, or left ventricular ejection fraction (LVEF) between the 2 groups were observed. For RV structure, only RVTD showed a difference. This illustrated that patients in these 2 groups were similar with regard to the hemodynamic and structural changes, which included enlargement of the right heart, broadening of the main pulmonary artery and left and right pulmonary artery branch, regurgitation of the tricuspid valve, and elevation of systolic pulmonary artery pressure. It is difficult to discern the severity of the disease only from 2D images. The 2010 ASE Guidelines for the Echocardiographic Assessment of the Right Heart in Adults proposed that a number of conventional echocardiographic parameters including TAPSE, s′, FAC, and MPI that can be readily assessed with high reliability and reproducibility should be considered in the diagnosis of PH.^[21]^ Our research divided patients into 2 groups according to RV LPSS and defined −19% as the cut-off value. We found that the RV function parameters, such as s′, TAPSE, RIMP, FAC, a′, and e′/a′, were significantly different between 2 groups. Patients with RV LPSS ≥−19% had a worse RV function. Haeck et al^[12]^ found that patients with a RV LPSS ≥–19% had significantly worse New York Heart Association functional class and lower TAPSE than their counterparts, and that RV LPSS was a significant determinant of all-cause mortality. It is well known that myocardial strain describes the extent of myocardial deformation. RV LPSS is defined as the percentage of myocardial shortening relative to the original length and is conventionally presented as a negative value. Therefore, the more negative the value of RV LPSS, the more preserved is the shortening. In the current study, the longitudinal myocardium of PH patients with RV LPSS ≥−19% was damaged more severely with respect to RV systolic and diastolic function.

Puwanant et al^[22]^ conducted a study on precapillary PH patients by using 2D-STI and found that a chronical increase in RV pressure directly impacts RV longitudinal systolic deformation. Increased RV pressure further affects interventricular septal and LV geometry, thus impairing LV torsion, segmental longitudinal strain, and circumferential strain.^[22]^ Sachdev et al^[23]^ found that pulmonary arterial hypertension patients exhibited a reduced RV systolic strain. Moreover, an RV free wall strain less than 12.5% was linked to a higher degree of disease progression within 6 months, and it was also predictive for 1-, 2-, 3-, and 4-year mortality. After adjustment for age, sex, PH cause, and functional classification, patients exhibited a 2.9-fold higher mortality rate per 5% absolute decline in RV free wall strain at 1 year.^[23]^ In a long-term follow-up study on patients with PH, Motoji et al^[24]^ found that patients with an RV-free >19.4% suffered from fewer cardiovascular events than those with an RV-free ≤19.4%. What is more, an RV-free ≤19.4% combined with a TAPSE <16 mm was correlated with the highest incidence of cardiovascular events.^[24]^

Our study indicated that RV LPSS was well correlated with TAPSE, FAC, RIMP, and s′ and thus, RV LPSS could reflect the degree of reduction in right heart function of PH patients. Li et al^[25]^ indicated that RV global longitudinal strain was more often altered in patients with severe PAH compared with mild PAH, and that global longitudinal strain and global longitudinal strain rate were associated with CMR-derived RVEF. Sunbul et al^[26]^ appraised RV function in CTEPH patients and discussed the association with activity tolerance by measuring RV longitudinal deformation using 2D-STI. They illustrated that patients with a shorter six-minute walk test (6MWT) distance exhibited a significantly larger RV but had a greatly lower RV fractional area change and higher myocardial performance index. Also, these patients had significantly lower RV basal-lateral strain and strain rate assessments compared with those with longer 6MWT distances. In addition, these patients exhibited lower RV basal-septal, mid-lateral, and global strain measures.^[26]^ In a previous study, RV LPSS was used to detect RV dysfunction in patients with severe PH, as well as early RV dysfunction in patients with mild PH.^[27]^ Consistent with the above finding, our study demonstrated the superiority of RV LPSS for uncovering severe PH (≥45 mm Hg) over the conventional parameters such as RIMP, TAPSE, s′, and RV FAC.

Pressure overload in PH leads to changes in the RV free wall that eventually result in RV failure. Hill et al^[28]^ suggested that the biomechanical and microstructural changes of the RV free wall myocardium should be used in future computational models, which may eventually be used to predict the response to altered hemodynamic states. Ventricular interdependence is defined as the size, shape, and compliance of 1 ventricle affecting the function of the other ventricle. The existence of an interventricular septum, involving 2 ventricles, limits expansion through the restricted pericardium, and RV increases load conditions, which adapts to the characteristics of the related cardiovascular system. Ricardo et al showed that impaired LV contractility in patients with PAH as assessed by speckle tracking strain was independent of ventricular septal involvement.^[29]^

## 5 Study limitations

Several limitations present in our study need to be pointed out. First, echocardiographic studies and RHC were not performed simultaneously. Second, we did not include control subjects with an mPAP <25 mm Hg. Third, because RHC is an invasive examination, its clinical application has indications, and not all patients could undergo the examination. Fourth, PVR is an important index of pulmonary hemodynamics as reflected in the pulmonary circulation pressure flow relationship. Therefore, the elevation of PVR is also considered a generalized pulmonary arterial hypertension. However, our study included several types of PH and demonstrated the issue of heterogeneity to some degree. Thus, elevated PVR was not considered in our study. In our future study, we will increase the sample size to make a more comprehensive analysis between the subgroups.

## 6 Conclusions

Distinguishing the degree of RV dysfunction by 2D-STI may help physicians to determine the state of cardiac function and the degree of PH in patients, which will offer a basis for the clinical diagnosis and treatment of PH.

## References

[1] Galie N Humbert M Vachiery JL 2015 ESC/ERS Guidelines for the diagnosis and treatment of pulmonary hypertension: The Joint Task Force for the Diagnosis and Treatment of Pulmonary Hypertension of the European Society of Cardiology (ESC) and the European Respiratory Society (ERS): Endorsed by: Association for European Paediatric and Congenital Cardiology (AEPC), International Society for Heart and Lung Transplantation (ISHLT). Eur Heart J 2016;37:67–119. 26320113 10.1093/eurheartj/ehv317

[2] Hoeper MM Lee SH Voswinckel R Complications of right heart catheterization procedures in patients with pulmonary hypertension in experienced centers. J Am Coll Cardiol 2006;48:2546–52. 17174196 10.1016/j.jacc.2006.07.061

[3] Sato T Tsujino I Ohira H Validation study on the accuracy of echocardiographic measurements of right ventricular systolic function in pulmonary hypertension. J Am Soc Echocardiogr 2012;25:280–6. 22230250 10.1016/j.echo.2011.12.012

[4] Schiller NB Kwan DM The Tei index as an expression of right ventricular impairment and recovery: investment grade or subprime? JACC Cardiovasc Imaging 2009;2:150–2. 19356548 10.1016/j.jcmg.2008.11.006

[5] Grignola JC Gines F Guzzo D Comparison of the Tei index with invasive measurements of right ventricular function. Int J Cardiol 2006;113:25–33. 16325940 10.1016/j.ijcard.2005.10.012

[6] Ostenfeld E Carlsson M Shahgaldi K Manual correction of semi-automatic three-dimensional echocardiography is needed for right ventricular assessment in adults; validation with cardiac magnetic resonance. Cardiovasc Ultrasound 2012;10:1. 22226082 10.1186/1476-7120-10-1 PMC3398276

[7] Jategaonkar SR Scholtz W Butz T Two-dimensional strain and strain rate imaging of the right ventricle in adult patients before and after percutaneous closure of atrial septal defects. Eur J Echocardiogr 2009;10:499–502. 19155264 10.1093/ejechocard/jen315

[8] Pirat B McCulloch ML Zoghbi WA Evaluation of global and regional right ventricular systolic function in patients with pulmonary hypertension using a novel speckle tracking method. Am J Cardiol 2006;98:699–704. 16923465 10.1016/j.amjcard.2006.03.056

[9] Dragulescu A Mertens LL Developments in echocardiographic techniques for the evaluation of ventricular function in children. Arch Cardiovasc Dis 2010;103:603–14. 21147445 10.1016/j.acvd.2010.09.004

[10] Galie N Hoeper MM Humbert M Guidelines for the diagnosis and treatment of pulmonary hypertension: the Task Force for the Diagnosis and Treatment of Pulmonary Hypertension of the European Society of Cardiology (ESC) and the European Respiratory Society (ERS), endorsed by the International Society of Heart and Lung Transplantation (ISHLT). Eur Heart J 2009;30:2493–537. 19713419 10.1093/eurheartj/ehp297

[11] Meris A Faletra F Conca C Timing and magnitude of regional right ventricular function: a speckle tracking-derived strain study of normal subjects and patients with right ventricular dysfunction. J Am Soc Echocardiogr 2010;23:823–31. 20646910 10.1016/j.echo.2010.05.009

[12] Haeck ML Scherptong RW Marsan NA Prognostic value of right ventricular longitudinal peak systolic strain in patients with pulmonary hypertension. Circ Cardiovasc Imaging 2012;5:628–36. 22875884 10.1161/CIRCIMAGING.111.971465

[13] Malenfant S Neyron AS Paulin R Signal transduction in the development of pulmonary arterial hypertension. Pulm Circ 2013;3:278–93. 24015329 10.4103/2045-8932.114752 PMC3757823

[14] Tanabe N Taniguchi H Tsujino I Multi-institutional retrospective cohort study of patients with severe pulmonary hypertension associated with respiratory diseases. Respirology 2015;20:805–12. 25828844 10.1111/resp.12530

[15] Brewis MJ Church AC Johnson MK Severe pulmonary hypertension in lung disease: phenotypes and response to treatment. Eur Respir J 2015;46:1378–89. 26293503 10.1183/13993003.02307-2014

[16] Fukazawa K Poliac LC Pretto EA Rapid assessment and safe management of severe pulmonary hypertension with milrinone during orthotopic liver transplantation. Clin Transplant 2010;24:515–9. 20002632 10.1111/j.1399-0012.2009.01119.x

[17] Lopez-Meseguer M Berastegui C Monforte V Inhaled iloprost plus oral sildenafil in patients with severe pulmonary arterial hypertension delays the need for lung transplantation. Transplant Proc 2013;45:2347–50. 23953548 10.1016/j.transproceed.2013.03.040

[18] Gall H Sommer N Milger K Survival with sildenafil and inhaled iloprost in a cohort with pulmonary hypertension: an observational study. BMC Pulm Med 2016;16:5. 26753921 10.1186/s12890-015-0164-2 PMC4709958

[19] Biswas M Sudhakar S Nanda NC Two- and three-dimensional speckle tracking echocardiography: clinical applications and future directions. Echocardiography 2013;30:88–105. 23297852 10.1111/echo.12079

[20] Jing Z Jianchang C Weiting X Comparison of left atrial function in healthy individuals versus patients with non-ST-segment elevation myocardial infarction using two-dimensional speckle tracking echocardiography. Cardiovasc J Afr 2013;24:154–60. 24217161 10.5830/CVJA-2013-011 PMC3748444

[21] Rudski LG Lai WW Afilalo J Guidelines for the echocardiographic assessment of the right heart in adults: a report from the American Society of Echocardiography endorsed by the European Association of Echocardiography, a registered branch of the European Society of Cardiology, and the Canadian Society of Echocardiography. J Am Soc Echocardiogr 2010;23:685–713. 20620859 10.1016/j.echo.2010.05.010

[22] Puwanant S Park M Popovic ZB Ventricular geometry, strain, and rotational mechanics in pulmonary hypertension. Circulation 2010;121:259–66. 20048214 10.1161/CIRCULATIONAHA.108.844340 PMC2846516

[23] Sachdev A Villarraga HR Frantz RP Right ventricular strain for prediction of survival in patients with pulmonary arterial hypertension. Chest 2011;139:1299–309. 21148241 10.1378/chest.10-2015

[24] Motoji Y Tanaka H Fukuda Y Efficacy of right ventricular free-wall longitudinal speckle-tracking strain for predicting long-term outcome in patients with pulmonary hypertension. Circ J 2013;77:756–63. 23220860 10.1253/circj.cj-12-1083

[25] Li Y Xie M Wang X Right ventricular regional and global systolic function is diminished in patients with pulmonary arterial hypertension: a 2-dimensional ultrasound speckle tracking echocardiography study. Int J Cardiovasc Imaging 2013;29:545–51. 22956022 10.1007/s10554-012-0114-5

[26] Sunbul M Kepez A Kivrak T Right ventricular longitudinal deformation parameters and exercise capacity: prognosis of patients with chronic thromboembolic pulmonary hypertension. Herz 2014;39:470–5. 23740084 10.1007/s00059-013-3842-y

[27] Ikeda S Tsuneto A Kojima S Longitudinal strain of right ventricular free wall by 2-dimensional speckle-tracking echocardiography is useful for detecting pulmonary hypertension. Life Sci 2014;111:12–7. 25064826 10.1016/j.lfs.2014.06.024

[28] Hill MR Simon MA Valdez-Jasso D Structural and mechanical adaptations of right ventricle free wall myocardium to pressure overload. Ann Biomed Eng 2014;42:2451–65. 25164124 10.1007/s10439-014-1096-3 PMC4241140

[29] de Amorim Correa R de Oliveira FB Barbosa MM Left ventricular function in patients with pulmonary arterial hypertension: the role of two-dimensional speckle tracking strain. Echocardiography 2016;33:1326–34. 27460782 10.1111/echo.13267

